# Lighting Strikes Twice: Recurrent Guillain–Barré Syndrome (GBS) after Influenza Vaccination

**DOI:** 10.1155/2021/6690643

**Published:** 2021-02-03

**Authors:** Gregory Griffin, Brittany Cunningham, Jonathan M. Beary, Yonatan Spolter, Richard Gandee, Christopher R. Newey

**Affiliations:** ^1^Cleveland Clinic Akron General, Neurological Institute, Akron General Avenue, Akron, OH 44307, USA; ^2^AT Still University-Missouri Campus, Neurobehavioral Sciences, 800 West Jefferson Avenue, Kirksville, MO 63501, USA; ^3^Cleveland Clinic, Neurological Institute, 9500 Euclid Avenue, Cleveland, OH 44195, USA

## Abstract

Guillain–Barré syndrome (GBS) is a rare acute demyelinating syndrome of the peripheral nervous system that is commonly preceded by infection. Vaccinations have also been associated with an increased incidence of GBS, though the risk is low. Caution with revaccination is recommended in patients with a history of GBS. Risks of revaccination compared with the risks of influenza complications should be considered. Patients who experience GBS after vaccination have not been shown to have an increased incidence of recurrent GBS after the influenza vaccine, though evidence is limited. We report a case of recurrent GBS in a patient following the influenza vaccine.

## 1. Introduction

Guillain–Barré syndrome (GBS) is a rare acute demyelinating syndrome of the peripheral nervous system [1]. It is classically characterized by ascending muscle weakness with loss of reflexes that progresses on average over five to ten days. Cerebrospinal fluid (CSF) analysis typically demonstrates an albuminocytologic dissociation (i.e., high CSF protein and normal cell counts) [2]. In severe cases, respiratory failure may occur requiring mechanical ventilation. Annually, there are 0.4–4.0 cases of GBS per 100,000 people. The incidence increases throughout adulthood being the highest in persons older than 75 years of age [3].

GBS is typically preceded by a nonspecific viral prodrome. Often, the offending pathogen is not identified. It has been shown to be associated with *Campylobacter jejuni*. Influenza infection and other upper respiratory tract infections have also been associated with an increased risk of GBS. The association between GBS and the influenza vaccine was first noted during the 1976 swine influenza season [3]. During this season, the cited risk of GBS was one additional case per 100,000 persons vaccinated [3]. The risk was highest within two to three weeks after receiving the vaccine. More recently, the 2009 pH1N1 vaccine was evaluated due to its swine origin [3]. Analysis suggests that with this strain of vaccine, the risk of GBS was 1–3 cases per 1 million persons vaccinated [3]. Overall, the association is approximated at 1-2 additional cases of GBS per 1 million persons vaccinated [3].

We present a case of recurrent GBS in a patient who received the influenza vaccination with a prior history of GBS following influenza vaccination five years prior.

## 2. Case

A 64-year-old male with a history of remote right middle cerebral artery (MCA) stroke and left hemiplegia was transferred from an outside hospital for ascending weakness of the right hemibody, areflexia, and acute respiratory failure requiring intubation. In 2015, he required 18 days of mechanical ventilation for GBS following influenza vaccination. At that time, he was treated with intravenous immunoglobulin (IVIG) and plasma exchange (PLEX) and made full recovery. He was eventually discharged to a long-term acute care hospital.

**Figure 1:**
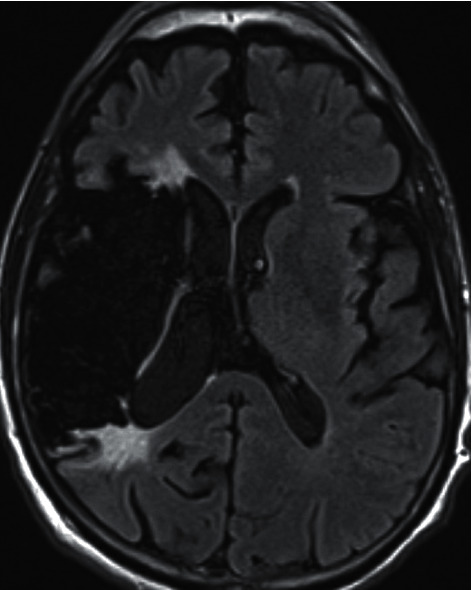
Magnetic resonance imaging (MRI). Fluid-attenuated inversion recovery (FLAIR) sequence shows the encephalomalacia from the right middle cerebral artery (MCA) infarct.

**Figure 2:**
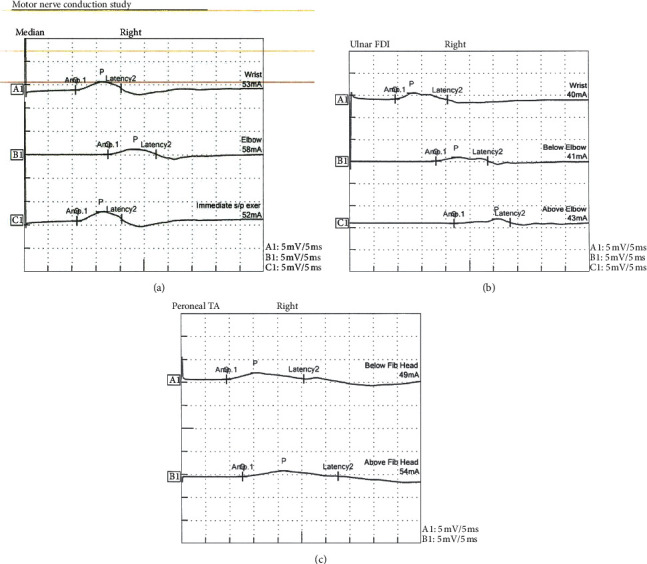
Nerve conduction study (NCS). Prolonged motor latency and reduced compound motor action potential (CMAP) amplitudes are noted and are shown in the median (a), ulnar (b), and peroneal (c) nerves.

The patient received the 2020 influenza vaccine approximately five weeks prior to admission to an outside hospital. He developed paresthesias of the right hemibody and weakness that progressed to inability to feed himself and ultimately respiratory failure 48 hours later. Imaging of the neuraxis showed the prior right MCA stroke (Figure 1). Initial evaluation in the neurosciences intensive care unit found him to be alert. His negative inspiratory pressure (NIF) was −5 cm H_2_O (normal > 60 cm H_2_O) and right upper and lower extremity power 0/5 with 0/4 reflexes. He was on apixaban for atrial fibrillation; thus, initial lumbar puncture was deferred while waiting for clearance of the medication. Given his history and examination, PLEX was started. He received 5 sessions over a period of 8 days. Lumbar puncture was performed and showed normal cell counts with elevated protein (108 mg/dl (normal 15–45 mg/dl)). Nerve conduction study (NCS) showed prolonged motor distal latencies and severely slowed conduction velocities with reduced compound motor action potential (CMAP; Figure 2). Sural sensory response was normal. *F* waves were absent. Electromyography (EMG) showed fibrillation potentials (+1) in the right deltoid along with severely decreased recruitment in the right triceps and right tibialis anterior. These diagnostic tests were consistent with GBS. He continued to improve clinically. By day seven, right upper extremity strength had improved to 3+/5 and lower extremity at least 3−/5 in proximal muscle groups. Patellar muscle stretch reflex improved to 1+/4 but Achilles reflex remained 0/4. He was successfully extubated and eventually transferred to a long-term acute care hospital for anticipated long-term rehabilitation and treatment.

## 3. Discussion

We report a case of recurrent GBS. In both incidences, separated by approximately five years, the influenza vaccine was given within 6 weeks prior to symptom onset. GBS is suspected to be immune-mediated in which antibodies respond to antigens and cross-react with nerve ending antigens, resulting in ascending weakness, areflexia, and in serious cases such as respiratory failure, autonomic instability, and death. Infection prior to onset of GBS is common; however, the specific preceding infection is unknown in up to 60% of cases. Data supporting this molecular mimicry hypothesis with specific infectious causes are limited [3]. The prognosis following GBS can be poor with up to 20% of patients remaining severely disabled and mortality in up to 5% of patients. If a patient is unable to walk without assistance, immunotherapy is recommended. Treatment with intravenous immune globulin (IVIG) within 2 weeks of onset is as effective as plasma exchange. IVIG is typically given at 0.4 g/kg bodyweight daily for 5 days or at 2 g/kg bodyweight given in 2 days. PLEX is often performed with a total of 5 exchanges over seven to ten days. The use of PLEX after IVIG is not superior to either therapy alone [2]. There is no role for steroids in treating patients with GBS [4].

There may be an increased risk for developing GBS from pandemic influenza vaccinations (such as H1N1) compared to seasonal vaccination [3, 5]. In several GBS variants, autoantibodies to gangliosides and related glycolipids, including GM1, GD1a, GD1b, and GQ1b, have been described. It is theorized that there is a pathogenetic role involving antibodies to gangliosides after vaccination [6].

Another review aimed to describe vaccination and GBS recurrence in patients with a history of GBS [7]. A total of 550 patients with a history of GBS were identified. Eighteen of these patients initially had onset after influenza vaccine. Two of these patients were revaccinated with the influenza vaccine and did not experience recurrence. In this cohort, 989 vaccines were given to 279 patients. The most common vaccine administered was the influenza vaccine (*n* = 107). Of 550 patients with GBS, 6 individuals had recurrent GBS symptoms. However, there were no episodes of recurrent GBS after influenza vaccine [7]. This information has been recently disputed. In a large, nested case-control study over 4 years in three Chinese cities, the occurrence of GBS following vaccination of any kind was studied [8]. It was found that the odds ratio (OR) for GBS occurrence to be 1.09 (95% CI 0.88–1.32) in adult patients within 180 days following vaccination [8]. The odds ratio for the recurrence of GBS after vaccination was found to be 1.18 (95% CI 0.49–2.65) [8]. In other words, the authors highlight that there was no evidence of an increased risk of GBS and its recurrence within 180 days following vaccination of any kind, including influenza vaccination [8]. If there is recurrence of GBS, neurological symptoms in subsequent episodes are often similar, while the severity of the symptoms and the nature of the preceding infections can differ in individual patients [9].

A case series of patients with recurrent GBS sought to determine if symptoms or proceeding infection was similar in patients with recurrent GBS [9]. Thirty-two patients had a total of 81 episodes of GBS [9]. Clinical symptoms were similar in recurrent episodes in individual patients [9]. However, there was noted variability in preceding infection and severity of symptoms [9]. Patients with recurrent GBS were younger at a mean age of 34.2 years vs. nonrecurrent patients of 46.9 years (*p*=0.001). Additionally, patients with recurrent GBS more often had Miller Fisher syndrome with the classic clinical triad of ataxia, ophthalmoplegia, and areflexia [9]. Notably, the patient described in our case did not meet all of these characteristics. He was older and had a classic GBS presentation. His presentation, however, was milder than his initial GBS. There likely is a genetic or immunologic host susceptibility that plays a role in recurrent GBS [9].

The Advisory Committee on Immunization Practices currently notes a precaution for revaccination in patients with a history of GBS after an influenza vaccine [4]. The committee also states that influenza vaccination may outweigh the possible risk for certain patients who have a history of GBS within six weeks of influenza vaccine if they are at higher risk for severe complications from influenza [4]. Although recurrence risk is documented to be low, clear risk factors for recurrent GBS have not been consistently defined in the literature. Further studies are needed to determine which patients are at the highest risk for recurrent GBS.

## 4. Conclusion

The risk of GBS following vaccination is low at one to three cases per million patients vaccinated. Additionally, the incidence of GBS recurrence after vaccines appears low but has not been well described. However, despite the low incidence, it is important to carefully weigh risks and benefits of revaccination.

## Data Availability

The data analyzed during the current case report are available from the corresponding author upon request.
